# Vaginal birth after caesarean section: why is uptake so low? Insights from a meta-ethnographic synthesis of women's accounts of their birth choices

**DOI:** 10.1136/bmjopen-2015-008881

**Published:** 2016-01-08

**Authors:** Mairead Black, Vikki A Entwistle, Siladitya Bhattacharya, Katie Gillies

**Affiliations:** 1 Division of Applied Health Sciences, University of Aberdeen, Aberdeen Maternity Hospital, Aberdeen, UK; 2 Division of Applied Health Sciences, University of Aberdeen, Aberdeen, UK

**Keywords:** OBSTETRICS, PUBLIC HEALTH, PERINATOLOGY

## Abstract

**Objective:**

To identify what women report influences their preferred mode of birth after caesarean section.

**Design:**

Systematic review of qualitative literature using meta-ethnography.

**Data sources:**

Medline, EMBASE, ASSIA, CINAHL and PsycINFO (1996 until April 2013; updated September 2015). Hand-searched journals, reference lists and abstract authors.

**Study selection:**

Primary qualitative studies reporting women's accounts of what influenced their preferred mode of birth after caesarean section.

**Data extraction and synthesis:**

Primary data (quotations from study participants) and authors’ interpretations of these were extracted, compared and contrasted between studies, and grouped into themes to support the development of a ‘line of argument’ synthesis.

**Results:**

20 papers reporting the views of 507 women from four countries were included. Distinctive clusters of influences were identified for each of three groups of women. Women who confidently sought vaginal birth after a caesarean section were typically driven by a long-standing anticipation of vaginal birth. Women who sought a repeat caesarean section were strongly influenced by distressing previous birth experiences, and at times, by encouragement from social contacts. Women who were more open to information and professional guidance had fewer strong preconceptions and concerns, and viewed a range of considerations as potentially important.

**Conclusions:**

Women's attitudes towards birth after caesarean section appear to be shaped by distinct clusters of influences, suggesting that opportunities exist for clinicians to stratify and personalise decision support by addressing relevant ideas, concerns and experiences from the first caesarean section birth onwards.

**Strengths and limitations of this study**

- Meta-ethnographic methods ensured sensitivity to contextual factors surrounding the influences reported by women planning birth after caesarean section.
- The contextual factors that were taken into consideration included the circumstances under which women were recruited and interviewed, and the timing of the interventions or exposures that influenced their views.
- The iterative process of reciprocal translation of study findings facilitated a higher level of understanding than previous mixed-method review methodology has allowed.
- The focus on women's perspectives is consistent with woman-centred approaches to care, but this review did not consider the views of health professionals and family.
- The identification of clustering of influences was robust to ‘testing back the fit’ which confirmed that primary authors’ interpretations supported the synthesis ‘line of argument’.

## Introduction

Caesarean section (CS) births are described as being at epidemic levels across middle-income and high-income countries.^1^ ^2^ One in three babies in the USA are born by CS.^1^ South American rates of CS exceed 50% in many areas, with over 70% of births in private healthcare settings being by CS.^3^ ^4^ Concern to reduce overall rates of CS is in tension with efforts to promote patient choice, as women themselves often request this mode of birth.^5^

The greatest contribution to current high rates of CS comes from repeat CS procedures.^6^ Worldwide rates of vaginal birth after CS (VBAC) have dropped dramatically in recent years. Between 1999 and 2002, US VBAC attempts fell from 48.3% in 2000 to 30.7% in 2002, with 73.4% of VBAC attempts being successful.^7^ The UK saw actual VBAC rates fall from 45.9% in 1988 to 36% between 2004 and 2011.^8^ ^9^ Health service support for VBAC diminished after retrospective data published in 1996 favoured the maternal safety profile of repeat CS.^10^ Although more evidence for the relative safety of VBAC has emerged in recent years,^11^ and efforts have been made to increase VBAC attempts, rates have never fully recovered.^12^ ^13^

Enthusiasm to reduce rates of CS stems from policy concerns about the relatively high financial costs and the greater maternal morbidity and mortality of CS when compared with vaginal birth.^14^ It can also be linked to broader concerns about unnecessary medical intervention (too much medicine).^15^ However, the costs and harms that are evident when CS is considered at a population level are much less apparent at the level of individual women. Absolute rates of serious morbidity from CS are low,^2^ ^16^ and there is little evidence that women themselves regret CS when they have requested this mode of birth.^17^ At the same time, potential benefits of CS can often be identified for (and by) individual women.^18^ Population data suggest that an increase in rates of CS does not contribute to parallel improvements in neonatal outcomes.^19^

Broad policy consensus in high-income countries supports offering women who become pregnant after CS a choice between repeat CS and attempting VBAC, unless clinical circumstances or available services preclude this (eg, when a high risk of CS scar rupture contraindicates VBAC).^12^ ^16^ ^20^ UK guidance outlines which risks (including probabilities) should be discussed by women and health professionals before agreeing on the planned mode of birth by 36 weeks gestation.^20^ Although probabilistic information about the physical health outcomes of VBAC and repeat CS might seem to support VBAC, the introduction of decision support interventions in the latter part of pregnancy after CS has made little difference to women's choices.^21^ ^22^ There are several plausible explanations for this, including the likelihood that decision-making is influenced by a much broader range of cultural values and social and emotional considerations than are addressed through existing decision support. It is known, for example, that some women have a strong desire to experience vaginal birth,^23^ ^24^ and that some fear dissatisfaction if they choose VBAC but their attempt fails.^25–27^ However, the insights that have emerged from studies, to date, have been somewhat fragmented. A more comprehensive and nuanced understanding of the complex range of influences on women's decisions is needed to support informed ethical judgements about efforts either to reduce rates of CS or to support women's decision-making. Development of public health policy and clinical practice would benefit from as robust as possible an understanding of the diverse perspectives that women bring to decisions about mode of birth following a previous caesarean, as would debate about what range of options, information, advice and decision support could be appropriately provided by health services. To address this need, we aimed to identify, contextualise and synthesise an understanding of the reasons why women prefer VBAC or elective repeat CS (ERCS).

## Methods

A systematic literature search and meta-ethnography was conducted. The seven steps of meta-ethnography described by Noblit and Hare, as listed in box 1, were followed to synthesise the available primary research studies.^28^

Box 1 **Meta-ethnography steps as described by Noblit and Hare^28^**

- 1. Identify the research question
- 2. Identify relevant studies
- 3. Read the studies
- 4. Identify themes
- 5. Translate the findings of each study into those of the others
- 6. Synthesise the findings
- 7. Express the synthesis

A systematic search was conducted using Medline, EMBASE, ASSIA, CINAHL and PsycINFO in April 2013 (updated in September 2015) using multiple subject headings and free text key words relating to modes of birth and exploration of women's preferences or choices (the full search strategy for Medline is provided as online supplementary appendix 1, and further search strategies are available from the authors on request). Inclusion and exclusion criteria are outlined in table 1.

**Table 1:** Inclusion and exclusion criteria

Inclusion criteria	
Study population	Comprised or included an identifiable subgroup of women who have had at least one previous caesarean section
Study design	Primary research that included and clearly reported a qualitative element
Study findings	Included accounts of influences on preferred mode of birth after a previous caesarean section, from the women's perspectives Primary data provided relevant to the research question and target population of this synthesis
Language	Any; no language restrictions applied
Exclusion criteria	
Date of publication	Studies published before 1996.

Titles, abstracts and, where necessary, full papers were screened for potential eligibility. Inclusion and exclusion criteria were applied to full papers. Authors were contacted when only abstracts were published and studies appeared relevant. Three journals containing the greatest number of relevant studies in the 2013 search (*British Journal of Midwifery*, *International Journal of Nursing Practice*, and *BJOG*: an *International Journal of Obstetrics and Gynaecology*) were hand searched to identify any further relevant papers. High-quality translation of two abstracts and one full article was obtained. Quality assessment was performed using the Critical Appraisal Skills Programme checklist for qualitative studies^29^ to prompt reflection on study quality, but studies were not excluded on the basis of quality if they contained some qualitative data of value to our research question.

The key characteristics of included studies were extracted and summarised (see table 2). The studies were initially read individually, in chronological order, and relevant points from the primary data (first-order constructs) and the study authors’ descriptions and interpretations (second-order constructs) were extracted. First-order constructs were obtained from quotations from women reported in the ‘results’ section of each study, while second-order constructs (primary authors’ account and interpretation of their findings) were obtained from ‘results’ and ‘discussion’ sections. All first and second-order constructs were tabulated in the form of primary quotes, or exact author interpretations, to support the identification of key themes.

**Table 2:** Characteristics of included studies

ID number	Author	Year	Country	Study aim	Data collection method	Planned birth method at time of study	Participants (n)	Timing of interview
M1	Ridley^30^	2002	USA	Discover what influences women in the decision to deliver via VBAC	Interview (FTF)	VBAC	5	Postnatal (2–4/12)
M2	York^31^	2005	UK	Describe childbirth expectations, influences and knowledge in women who had experienced emergency CS and planned subsequent CS	Interview (FTF)	CS	10	Antenatal (third trimester)
M3	Liu^23^	2006	China	Investigate the decision factors involved and experience of women who had successful VBAC	Interview (FTF), researcher diary, field notes	VBAC	10	Postnatal (1–2/7)
M4*	Fenwick^18^	2006	Australia	Describe childbirth expectations, influences and knowledge in women who had experienced emergency CS and planned subsequent CS	Interview (T), field notes	CS	49	Pre-pregnancy, antenatal and postnatal (no limits)
M5	Emmett^32^	2006	UK	Explore women’s experience of decision-making regarding mode of delivery after having a previous CS	Interview (FTF)	VBAC and CS	21	Postnatal (2–8/12)
M6	Cheung^33^	2006	China	Understand Chinese women's perceptions and interpretations of their own CS decision- making, and to investigate how their negotiation with healthcare professionals may be improved	Interview (FTF), field notes	CS	52	Postnatal (1/52 or 8/12)
M7	Meddings^34^	2006	UK	Examine the lived experience of women who elected to attempt a vaginal birth following a previous CS delivery	Interview (FTF)*2	VBAC	8	Antenatal (>34/40) and postnatal (∼6/52)
M8	Moffat^35^	2007	UK	Prospectively explore women's decision-making regarding mode of delivery after a previous CS	Consultation observation, patient diaries, interview (FTF)	VBAC and CS	26	Antenatal (from 20/40) and postnatal(6/52)
M9*	Fenwick^36^	2007	Australia	Explore childbirth expectations and knowledge of women who had experienced a CS and would prefer a vaginal birth in a subsequent pregnancy	Interview (T)	VBAC	35	Pre-pregnancy, Antenatal and Postnatal (no limits)
M10	Farnworth^37^	2007	UK	Identify and describe factors which influence women making a choice regarding mode of delivery after a previous CS delivery in a UK setting, and to identify the role of the obstetrician in this process	Interview (FTF)	VBAC and CS	10	Antenatal (36/40)
M11	Cox^38^	2007	UK	Explore issues around the choices between VBAC and elective CS based on the nature and extent of the information women actually received when making a decision between elective CS and VBAC, the sources of that information, and its importance in terms of the influence it had on their decision	Interview (type not clear)	VBAC and CS	7	Postnatal (timing not clear)
M12	Farnworth^39^	2008	UK	Examine the impact of a decision support intervention designed for women choosing mode of delivery after one previous CS	Interview (FTF)	VBAC and CS	18	Antenatal (37/40)
M13†	McGrath^40^	2009(a)	Australia	Explore, from the mother's perspective, the decision-making experience with regard to subsequent birth choice for women who had delivered previously by CS	Interview (FTF)	CS	16	Postnatal (6/52)
M14†	McGrath^41^	2009(b)	Australia	Describe the perspective of mothers who underwent elective CS on risks associated with the delivery modes of VBAC and elective CS, and their experience discussing such risks with their health professionals	Interview (FTF)	CS	16	Postnatal (6/52)
M15	Goodall^42^	2009	UK	Explore women's perceptions of the role of health professionals in their decision regarding mode of delivery, following previous delivery by CS	Interview (FTF)	VBAC and CS	8	Antenatal (20–40/40)
M16	Frost^43^	2009	UK	Obtain the views of women on their experiences of decision-making about the method of delivery following a previous CS , and the role of decision aids in this process	Interview (FTF)	VBAC and CS	30	Antenatal (37/40), postnatal (6–8/52)
M17†	Phillips^24^	2009	Australia	Explore, from a phenomenological perspective, the reasons motivating women to try for or achieve VBAC	Interview (FTF)	VBAC	4	Postnatal (6/52)
M18†	McGrath^44^	2010(a)	Australia	Explore, from the mothers’ perspective, the process of decision-making about mode of delivery for a subsequent birth after a previous CS	Interview (FTF)	VBAC	4	Postnatal (6/52)
M19	David^45^ Originates from same study as	2010	Australia	Provide maternity healthcare providers with an increased understanding of, and insight into, the different information needs of this specific group of maternity care consumers.	Telephone log and field notes	VBAC	170	Antenatal (various gestations)
M20†	McGrath^46^	2010(b)	Australia	To focus on findings which recorded the frustration of women who valued a vaginal delivery but who delivered by CS	Interview (FTF)	CS	8	Postnatal (6/52)

*Originates from same study (M4 and M9).

†Originates from same study (M13, M14, M17, M18 and M20).

CS, caesarean section; FTF, face-to-face; M, manuscript; T, telephone; VBAC, vaginal birth after CS.

Searching was conducted by one author (MB), with input from an information specialist. Screening and identification of studies, followed by coding of constructs were conducted by two authors (one clinical (MB), one non-clinical (KG)) independently, with regular meetings to establish agreement. During these meetings, provisional third-order constructs (our interpretation of both primary authors’ interpretations and primary data) and key themes were identified. The third and fourth authors (VAE and SB) were involved in further development of these themes, having each reviewed a different sample of included studies.

The key interpretive aspect, step five of Noblit and Hare's approach, involved one author comparing and contrasting the constructs and themes that featured in the different studies in an iterative manner. The findings of each study were interpreted in light of each of the other relevant studies in turn. This allowed for detailed consideration of how study design and context could have shaped study findings (eg, which women were included and when they were interviewed in relation to their original CS and/or subsequent birth). During this process, third-order constructs were confirmed, and a line-of- argument synthesis developed. All four authors contributed to the development of the line of argument.

The potential for the clinical background of two authors (MB and SB) in particular to influence the findings was recognised from the outset. All team members’ interpretations and preconceptions were continually challenged and used in a constructive manner during discussions throughout the synthesis process to ensure that all reported perspectives were fairly considered, and that the line of argument developed was robust.

Following the updated search in September 2015, additional eligible papers were identified. Relevant findings were used to test the fit of the line of argument. This involved identification of first and second-order constructs (primary data and authors’ interpretations, respectively) in the additional papers, and analysing these for relevant themes of influence on birth preferences after CS. These themes were compared and contrasted with the content of the line of argument to assess the extent to which they appeared to ‘fit’ together or ‘conflict’ with one another.

## Results

**Figure 1:**
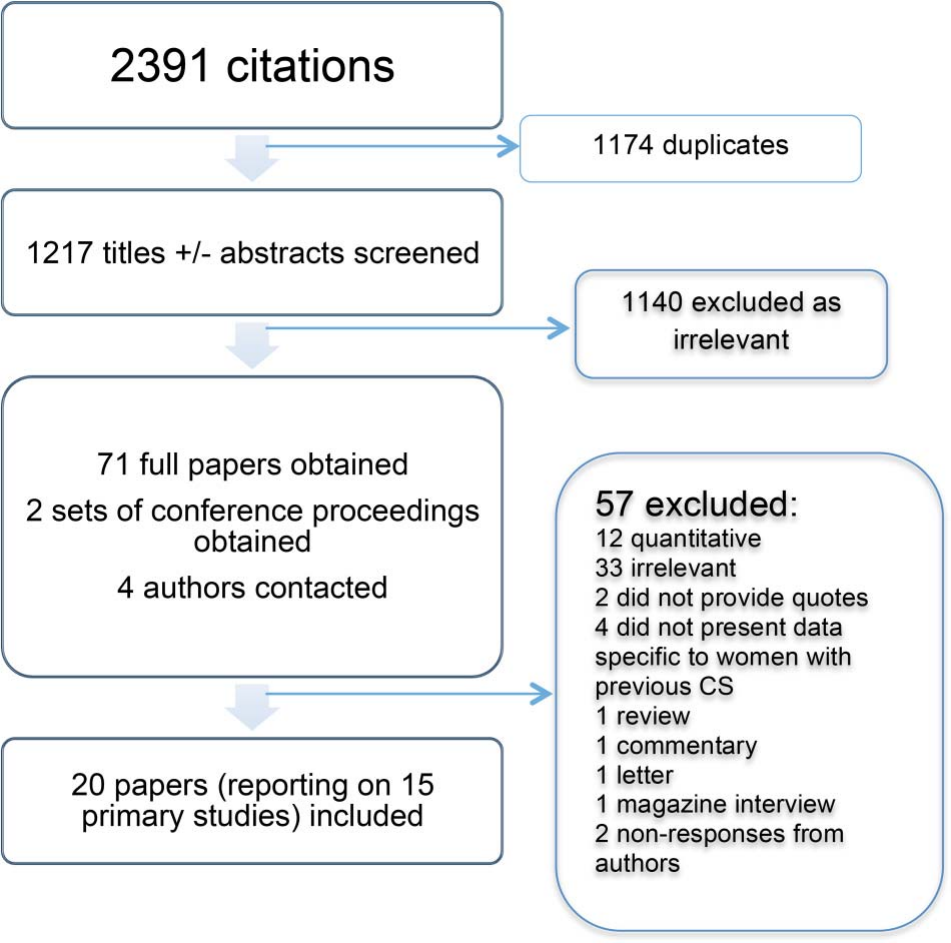
Flow diagram of search results’ caesarean section. CS, caesarean section.

The search results are outlined in figure 1. Of 2391 citations obtained in the original search, 1174 duplicates were excluded. Screening of 1217 titles and/or abstracts resulted in a further 1092 exclusions for lack of relevance; 71 full papers and two sets of conference proceedings were obtained, and attempts made to contact four authors, of which two were unsuccessful. A total of 57 titles lacked relevant primary data or were published before 1996 and were excluded. Twenty papers reporting from 15 primary studies were included following resolution of disagreement over eligibility of two papers.

The focus and key study characteristics for the 20 included papers are outlined in table 2.

The identified studies were conducted in four countries (UK, USA, China and Australia) and each included between 4 and 170 women, with findings from 507 women in total reported across the papers. Six papers reported on women who planned VBAC, four reported on women who planned ERCS, nine reported on both, and one reported on women who planned ERCS but would have desired VBAC in other circumstances.

Quality assessment of the papers is presented in online supplementary appendix 2. All papers had a clear statement of study aim which deemed qualitative methods to be appropriate. Common quality concerns included lack of information on: justification for the theoretical approach; lack of information about women who declined to take part; the interview guide used; and data saturation. Only one paper included a discussion of the potential for the researcher's role to influence the study's findings, although two further papers described involvement of a multidisciplinary team to perform the data analysis, mitigating the risk of dominance of a single interpretive perspective.

Our initial grouping of first and second-order constructs resulted in 40 subthemes. These were then categorised into six key themes which characterised the main kinds of consideration and features of decision-making processes that appeared to influence preferences for mode of birth. These themes were: long-standing anticipation of vaginal birth; responses to previous birth experiences (positive and/or negative); encouragement or dissuasion from influential people for either birth mode; fear or reassurance from risk-related information on VBAC; perceived net benefit or harm of birth options; and extent and nature of involvement in decision-making. As the labels suggest, several of these themes accommodate a spectrum of views or experiences.

### Key themes

The six key themes identified as shaping birth preferences after CS are illustrated with example data in table 3. Primary study participant quotes illustrating first-order constructs are displayed in bold text, and primary author interpretations illustrating second-order constructs are presented in italics.

**Table 3:** Key themes of influence on birth preferences after CS, with corresponding example data

Theme	Exemplary quote
Long-standing anticipation of vaginal birth	‘**Right from the start I wanted a natural delivery. All the women in my family just gave birth naturally and so I was very disappointed when it didn't work out that way for the first baby’** (M17) ‘*Despite their CS they still considered women's bodies were ‘designed’ to give birth vaginally’ (M9)*‘*Some of the study cases believed, due to their own notions, that there was only one way to feel like a real mother, ie. experiencing vaginal birth and the delivery pain in person. This was why they chose VBAC*’ (M3)
Responses to previous birth experience (positive and/or negative)	**‘If my body can't do it** [vaginal birth]**, why put myself and bub** [baby] **through all the stress and heartache’** (M13) ‘*Many of these women also expressed that the CS experience had made them feel powerless and helpless; ‘taking away total control*’’(M9) ‘**In the end we said, look, we're going to go with what we know. What we did first time worked out okay’ (M13)**
Encouragement or dissuasion from influential people for either birth mode	‘**they** [doctors] **said you can try normally, but they didn't seem very positive that it would work and I think they preferred me to have a caesarean’.** (M11) ‘*Horror stories’ and the knowledge and/or personal experience of friends also worked to reinforce their emerging view that CS was the safest birthing option’* (M4) ‘*..other sources of information were noted as mothers groups and/or playgroups.[where] ..sharing of knowledge ‘inspired’ them’* to pursue VBAC (M19)
Fear or reassurance from risk-related information on VBAC	**‘I like to gather as much information as I can about things and then make my own decisions from that’** (M17) ‘*A persistent theme appeared to be the lack of both local written information and professional opinion…this led the women to base their knowledge on a mixture of media, professional and personal sources’* (M2) ‘*Some women described feeling very sure about their preferred mode of delivery from the beginning of pregnancy and those women generally needed little in the way of decisional support*’ (M8).‘*Information and support gave women confidence in their decision, and ultimately, the power to own and justify the decision that they had made’* (M12) ‘**Oh yeah, the riskiest approach was to try a vaginal delivery. Yeah, no I wouldn't even have attempted it. And everything I read backed that up, yes.’ (M14)**
	‘**supposed to have all that stuff squeezed out and that's not done in a CS but it's probably less risky for the baby**’ (M4) ‘**About the biggest thing for me was the success rate.. . .There was more positive than negative.. . .. 80% of the women who tried it were able to do it’***.* (M1) ‘*When deciding whether to accept the VBAC or not, in most cases patients would first evaluate the advantages and disadvantages which included the recovery time after delivery, time of hospitalisation, potential harms to the mother and baby.’* (M3) ‘*women…considered CS a physical, emotional and lifestyle disruption that was risky and had the potential to cause harm to both mother and baby; separated them from their baby; and interrupted the postnatal period’* (M9)
Extent and nature of involvement in decision-making	‘**I was basically told they would prefer for me to try vaginal delivery but I could have a section if I really wanted’ (**M8**)** ‘**I feel every time I go and see the doctor or the midwife they keep talking about elective Caesareans…they keep finding reasons why I'll probably need an elective Caesarean so yeah it feels like choice is lot more limited this time’ (**M15**)** ‘*The important point is that the mothers who tried for a VBAC were clear and focused in their determination to own the decision-making process’ (M1)*

Primary study participant quotes are displayed in bold text and primary author interpretations are presented in italics.

CS, caesarean section; VBAC, vaginal birth after CS.

### Patterns of influence: a line of argument

We noted that some kinds of views and experiences (specific instances of the six key themes) tended to cluster together in support of the main birth preferences. These clusterings are discussed in the context of the line of argument we developed using the process of meta-ethnography to synthesise knowledge of influences on women's birth preferences after CS.

Women approaching a birth after a CS generally have either a clear preference for VBAC or ERCS, or a relatively open mind to either option. Although some studies by design included women from only one or two of these categories, looking across the studies, we were able to develop a line of argument to explain how their findings were related. In summary, the line of argument is that three distinctive clusters of influences support the three attitudinal positions that women adopt towards mode of birth after CS.

**Figure 2:**
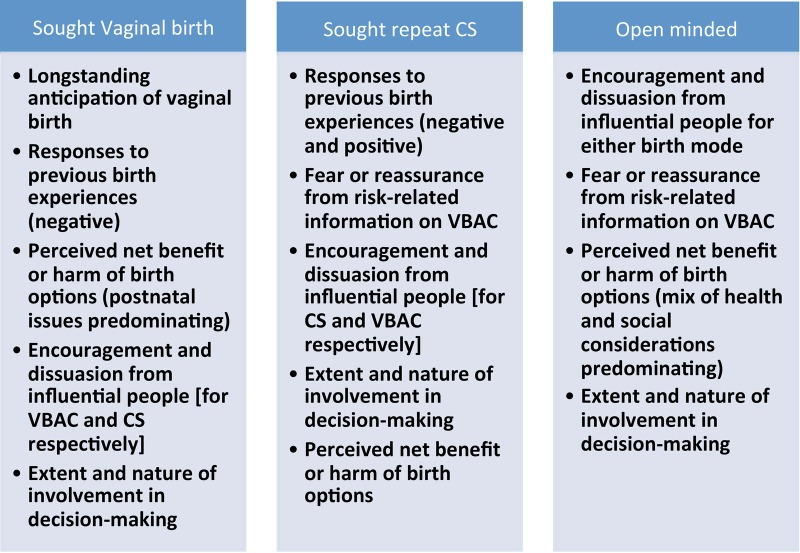
Summary attitudinal positions of women early in the pregnancy after CS and clusters of key influences acting on their eventual birth preferences. CS, caesarean section; VBAC, vaginal birth after caesarean.

The three positions and the distinctive influences on these are summarised in figure 2 and described below. We note that the influences could be operative from different times, and that some were significant before and around the first CS.

### Preferences for vaginal birth

Preferences for vaginal birth could be shaped by influences acting over a period of time, which for some women reached several years, and for many was linked to key events or periods of their lives. With respect to women's long-standing anticipation of vaginal birth, some women had a personal ambition to achieve vaginal birth that predated their first pregnancy and drove them to pursue VBAC (M17 and M3 (subject ID numbers)). This could act synergistically with negative responses to a previous birth experience. For example, unpleasant memories of the initial CS experience, particularly where women had felt a loss of control over that birth, led some women to view VBAC as a potentially life-enriching experience that met their ambitions and avoided further negative emotions (M1, M19, M9, M3, M8 and M14). This impression was often enhanced by interpregnancy social interaction with influential others, including women who provided encouragement by sharing accounts of their own positive VBAC experiences (M19). For some, the probability of successful VBAC was pivotal (M1 and M3).

Future considerations could also play an important role in the shaping of preferences for VBAC, as women considered implications beyond the birth itself when evaluating their expected net gain from VBAC. Several women believed that VBAC offered physiological benefits to physical and emotional health of themselves and their offspring, with particular emphasis on the facilitation of bonding and breastfeeding (M17 and M3). This was a particularly dominant issue among women who experienced breastfeeding difficulties after a previous planned CS, especially in those who had successfully breast fed their babies born vaginally in prior pregnancies (M3). The social benefits of being able to return to usual family roles and resume driving as soon as possible in the postnatal period were also cited as reasons for preferring to avoid CS particularly within UK study settings (M7, M8 and M9).

Further, influential people included health professionals who provided support, advice or encouragement in favour of VBAC. Women's perception of the extent to which they themselves should make the decision regarding planned mode of birth was important. Although some women, particularly in the UK and Australia, were confident about their right to decide how to plan the birth (M18, M7, M17 and M1), others judged any personal reasons they had in favour of ERCS to be unimportant or unjustified when considered in light of medical advice in favour of VBAC (M8).

### Preferences for ERCS

Response to the previous birth experience was the central theme among women who demonstrated a clear preference to have an ERCS. A previous emergency CS in labour appeared to lead many women to believe their bodies were incapable of vaginal birth (M8, M10 and M13). Some women sought an ERCS to actively avoid any possibility of a repeat emergency CS (M8, M10 and M13), while others feared the possibility of a recurrence of the factors which led to the previous CS. Others opted for ERCS on the grounds that it was a familiar and positive birth experience (M19, M5 and M6).

The previous birth and its outcome could also shape women's perceptions of the safety of VBAC (as outlined, it could lead to an assessment of net harm from planning VBAC), moderate the influence of social contacts (favouring those who encouraged ERCS and/or discouraged from planning VBAC) and limit the degree to which they felt they had a choice to make in the subsequent pregnancy (role in decision-making).

Safety concerns were described as particularly influential among some women in Australia who wished to avoid VBAC due to fear of the uterine scar ‘splitting’, or ‘rupturing’ during labour. This feeling dominated their preference for ERCS despite awareness of neonatal breathing problems being more common following this mode of birth (M4). Some women with a strong preference for VBAC had been influenced, sometimes powerfully, by family, friends and health professionals who recommended ERCS as a safer and more predictable mode of birth than VBAC (M13 and M4).

Ownership of choice, or lack of the same, appeared crucial in determining whether or not some women opted for ERCS. Many women perceived that their health professionals would prefer this option, and as such, that VBAC was not available to them (M15). Others choosing ERCS felt happy to exercise their preference as they had been positively encouraged to opt for the mode of birth that felt right for them (M5).

### Open-minded approach

Women who did not have a firm preference for either VBAC or ERCS appeared to be less strongly influenced by prior expectations about childbirth or by their previous birth experience than those who were more committed to one particular mode of birth. Influential others were apparently key to the decisions made in this context. These women valued and often actively sought the opinion of health professionals during their pregnancy, processed information on the options available and put considerable effort into weighing up the attributes of the birth options available to assess net benefit. An exception to this involved women who felt overwhelmed by the decision-making responsibility, and preferred to follow health professionals’ advice (M19, M8 and M18). Obstetricians, and, at times, midwives, appeared to have particular influence over women who were open to considering either mode of birth, even when women were not actively advised as to how to deliver, but perceived subtle signals that their health professional had a preference (M11). Some women said their choice should be based on information alone, rather than the input or opinions of others, recognising that other people are not necessarily impartial (M17).

### Robustness of findings

On ‘testing back the fit’ of our line of argument, we found that the clusters of influence we identified were consistent with the findings of each of the individual included studies, but that none of these studies included a broad enough mix of participants to have enabled the development of this level of understanding in isolation.

Further ‘testing’ of the line of argument was made possible by the publication of the three new studies identified in the update of the search conducted in 2015 which are summarised in table 4. Shorten *et al* analysed written text in which women explained their reasons for choosing either mode of birth after CS. They highlighted the significance of previous birth experience, safety concerns and speed of recovery along with health professionals’ preferences in shaping eventual decisions. Although they did not describe a clear distinction between the attitudinal groups, their findings were broadly supportive of the conclusions of this synthesis, with no evidence of confliction or contradiction.^47^ Kennedy *et al*^48^ performed an institutional ethnography exploring the complexity of choice around elective CS. This included interviews with women within the English National Health Service provider settings. The authors identified that women planning birth after CS negotiated with clinicians to reach a ‘comfortable compromise’ which facilitated a plan for VBAC that included adequate assurance of early recourse to CS if labour progress was suboptimal. This supports our findings of the crucial role of health professionals in influencing VBAC decisions by providing support for this option. Further author interpretation echoed our emphasis on the importance of predicted VBAC success in influencing women to aim for this mode of birth. Finally, the authors highlighted the desire for information among some women, providing an exemplary quote which supported our impression that women with an open mind to mode of birth after CS place great emphasis on the content, and in this case, quality of information accessed:

**Table 4:** Studies identified in the updated search which were used to ‘test the fit’ of the line of argument

Author	Year	Country	Study aim	Data collection method	Planned birth method at time of study	Participants (n)	Timing of data collection
Shorten^47^	2014	Australia	explore values and expectations that guide women during decision-making about the next birth after caesarean, and identify factors that influence consistency between women's choices and actual birth experiences	Written surveys and narrative accounts	VBAC and CS	187	36–37 weeks’ gestation and postnatal
Kennedy^48^	2013	UK	To explore the complexities of women's and clinicians’ choices around elective caesarean delivery	Interview (FTF) and consultation observations.	CS and vaginal birth (sample not restricted to birth after CS)	27 women, of whom three had VBAC and 19 had no history of prior CS. Previous obstetric history of 5 participants who underwent CS was not clear	Not specified; appears to span antenatal and postnatal period
Tully^49^	2013	UK	To document the circumstances in which caesarean section was deemed to be appropriate in one UK hospital through the eyes of the women and their partners experiencing the operative delivery of their infant	Interview (FTF)	Not applicable (postnatal)	115 women	Postnatal hospital stay

CS, caesarean section; FTF, face-to-face; VBAC, vaginal birth after CS.

When I was getting told about the 0.3% chance of a scar rupturing, you know, when I was asking people about how that statistic was arrived at no one could tell me, so I kept digging for more and more information, ‘and there's just not enough research, there's not enough studies that have been done, the women aren't in the same circumstances, they're not all in even one country, it's international, it's in under-developed countries, so you're pulling together these statistics from a complete diverse set of sample set, and how can you make judgements on what an individual's circumstances are going to be based on that? There's just not enough there's not enough information out there to be able to say you're going to be one of those statistics. (P108; woman pondering VBAC decision)

Tully and Ball^49^ presented findings of an interview study of 115 mothers recently delivered by CS over a 3-year period in England. Although minimal primary or secondary constructs related to birth after CS were presented, there was evidence that predicted VBAC success was important to women aiming for a vaginal birth, and that a negative previous birth experience drove women to seek control and predictability in the form of an ERCS. These observations are consistent with our findings, and no evidence of contradictory interpretations was identified.

## Discussion

### Summary of main findings

This study sought to answer the research question ‘What influences women's preferred mode of birth after previous caesarean section?’ We have identified distinct clusters of influences that tend to underpin the three main positions that pregnant women adopt towards modes of birth. After an initial CS, women tend to approach childbirth with one of three broad attitudinal positions meaning that they: (1) seek vaginal birth (2) seek repeat caesarean or (3) are open minded to consideration of either mode of birth. These positions reflect thought processes which are likely to evolve from at least as early as the primary CS, with some influential cultural norms in operation well before that time. A strong preference for VBAC appears to be driven by a belief that vaginal birth is ‘normal’ and has some intrinsic value. This belief is often accompanied by a keen desire to resume a normal life soon after vaginal birth. By contrast, a clear preference for ERCS from early in pregnancy can be driven by a previous negative experience of attempting but failing to achieve vaginal birth, and a positive emphasis on the predictability of ERCS. Finally, there are women who embark on their next pregnancy undecided about mode of birth. These women are more open to external influence: they appreciate the benefits of written information and personalised expert advice which they use to weigh up what they see as the advantages and disadvantages of their options. The recognition of these clusters of influences, according to attitude towards birth from early in the pregnancy after CS, is a novel finding made possible by looking across the range of relevant studies. Historical and contemporary studies, have highlighted influences on birth preferences after CS which resonate with those identified in this synthesis, but without identification of attitudinal groups or attention to the multiple influences and the ways these may vary over time.^27^ ^50^ ^51^ The importance of timing of influence has, however, been highlighted recently by prospective work which found that first-trimester preferences for either ERCS or VBAC persist by early in the third trimester in over 70% of women.^52^

### Benefits of a meta-ethnographic approach

Meta-ethnography enabled an interpretation of the available research that incorporated a sensitivity to the contextual factors surrounding the influences reported by specific groups of women planning birth after CS.^53^ Contextual factors considered included key time points at which influences took hold, fundamental study characteristics (setting; eligibility criteria; recruitment processes; timing of interviews; healthcare systems) and factors unique to individual women. These contextual considerations limit the likelihood that findings would be generalised inappropriately. The iterative process of reciprocal translation used to build on emergent themes facilitated a higher level of understanding than previous mixed-method review methodology has allowed, particularly that of quantitative work, where presence or absence of potential influences has been the focus.^25^ The clustering of influences identified within specific attitudinal groups provided clinically relevant insight into the nature of women's decision-making behaviour. In addition, the identification of clustering was considered robust in light of the ‘testing back the fit’, which confirmed that primary authors’ interpretations supported specific attitudinal clusters.

### Women's perspectives

The specific focus on *women's* perspectives on what influences birth preferences after CS complements the current focus on joint healthcare decision-making in which informed patients contribute to decisions which reflect their beliefs and preferences.^54^ This, therefore, provides insight which has maximal clinical application in settings where every effort should be made to ensure decisions about mode of birth after CS incorporate women's values and preferences. Given that health professionals have a variable level of input into shaping the eventual mode of birth, it is possible that consideration of health professionals’ perspectives may have further developed our understanding of the decision-making process.^55^ However, women's insights were considered central to achieving the goal of informing future efforts to optimise and support woman-centred planning of birth after CS.

### Clinical and research implications

#### Reflection on current practice

The strength of evidence supporting the first CS birth experience as a key influence on future birth preferences demands immediate attention. Women should be effectively supported in dealing with the unexpected and potentially traumatic nature of a primary CS. Efforts to promptly address any inaccurate perceptions of their CS birth events, and to provide personally specific information about the risks and benefits of future birth options could be made following the first CS, and be reiterated early in the pregnancy after CS. The findings of this synthesis suggest that women's concerns about serious maternal or offspring health risks (beyond those of CS scar rupture) are not important influences on their birth choices after CS. This is of particular interest because information currently provided by health professionals for women planning birth after CS focuses largely on these risks and clinical health considerations.^20^ Recognition of this mismatch between what women and health professionals prioritise should prompt health professionals to engage in discussion with women which allows identification of their main concerns and places sufficient emphasis on the psychological and social, as well as the physical health consequences of modes of birth after CS. The heterogeneity of influences on birth choices after CS demonstrated in this synthesis highlight why approaching all women planning birth after CS with, for example, the same decision support tool in the latter part of pregnancy, is unlikely to alter their prior attitudinal positions.

### Implications for future research and practice

**Figure 3:**
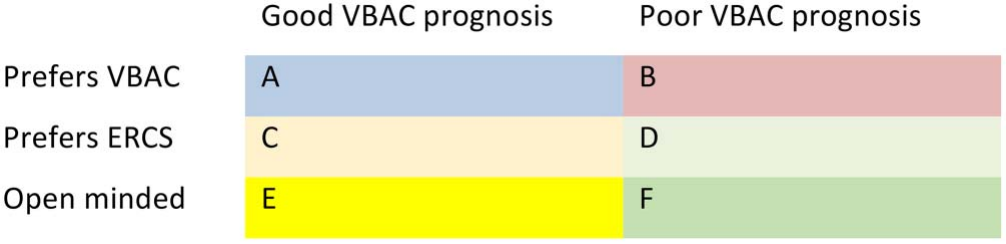
Table represents how women may be categorised according to their preferred mode of birth in early pregnancy and their prognosis for VBAC success’ VBAC, vaginal birth after caesarean; ERCS, elective repeat caesarean section.

Recognition of the diverse range of influences on, and attitudes towards birth after CS enables us to understand why decision support interventions have had limited effects on ERCS so far,^21^ ^22^ and opens up the possibility of a more targeted approach. We suggest that future interventions should aim to promote positive experiences of informed and shared decision-making, while minimising maternal and fetal morbidity, and avoiding unnecessary healthcare costs. Insights from this synthesis suggest that future strategies should ensure early consideration of women's concerns and preferences, and their likelihood of achieving good physical birth outcomes. Women may be broadly categorised in early pregnancy after CS as being in favour of either VBAC or ERCS, or being open to either option. At the same time, their prognosis for successful VBAC may also be assessed based on factors such as their age, body mass index and indication for previous CS.^8^ ^56^ To support high-quality decision-making and increase VBAC success rates, efforts could be made to ensure design of decision support which reflects women's prognosis for VBAC success and is sensitive to any early preferences regarding mode of birth after CS. The six main prognosis/preference categories are represented in figure 3.

Decision support for women may be delivered via conversations with health professionals, advice and information, including decision aids.^57^ Decision aids provide women with information about options relevant to their health status, while helping them to reflect and draw on their personal values. Previous research has demonstrated that use of some such tools in supporting birth choices after CS improved decision satisfaction but had minimal impact on VBAC rates.^22^ The lack of success in increasing VBAC rates may reflect that the tools that were tested were not tailored to women's early attitudes towards each birth mode, but instead delivered advice according to outcomes which women prioritised. Faced with a choice of surgery and less invasive options, decision aids have been shown to lead patients to choose conservative or less invasive treatments.^58^

In the context of planning birth after CS, decision aids might usefully be stratified according to predicted VBAC success and also be responsive to individual women's early preferences and priorities of mode of birth. It is likely to be particularly important to engage women who are open minded (groups E and F on figure 3), and women with a VBAC prognosis which is at odds with their preferred mode of birth (groups B and C in figure 3) by the second trimester, in conversations with health professionals, to ensure sufficient time to explore their views and discuss and allow them to consider their options. In such situations, a ‘consider a recommendation’ approach may be warranted, explaining why either ERCS or VBAC is recommended, but leaving sufficient scope and ensuring sufficient support for women to assess and discuss the recommendation before making their own mind up about it.^59^ In those pursuing VBAC despite a poor prognosis for success, there could be a discussion about criteria for conversion to CS, and adequate counselling in preparation for the possible psychological impact of such an outcome. Those in whom VBAC prognosis is in keeping with their preferred mode of birth (groups A and D in figure 3) might need less in the way of information, conversation and recommendations from health professionals, but their needs for information and reassurance about their decisions should not be neglected: balanced written information regarding the risks and benefits of both birth options, and clarification/confirmation of ongoing preferences are still likely to be important. As events unfold during subsequent pregnancies, ongoing communication and decision support for all women would need to be tailored to accommodate new clinical information, concerns and preferences, but a broad pathway identified following the first CS would ensure timely and relevant intervention to address modifiable influences.

## Conclusions

Forming a preference for repeat CS or VBAC is a dynamic process shaped by many influences which appear to cluster distinctively in the development of strongly held positions. Long-standing expectations of childbirth and perceptions of previous birth experiences appear particularly influential on VBAC and ERCS preferences, respectively. This suggests that early communication to discuss women's prospects for VBAC success and explore and discuss their attitudes towards future births may be valuable, and could perhaps start from as early as the first CS. This might help increase the proportion of women who approach birth after CS with an open mind, being receptive to written information, and the advice of health professionals. Our synthesis has highlighted why current care models involving provision of information in pregnancy after CS may not lead to the birth choices which could help reduce the unnecessary rate of CS. It suggests a need to address women's social and psychological concerns, and not just the currently recommended information, both to support women's autonomy in decision-making, and to address public health concerns about rising rates of clinically unnecessary CS.
